# Determining the Clinical Utility of 16S rRNA Sequencing in the Management of Culture-Negative Pediatric Infections

**DOI:** 10.3390/antibiotics11020159

**Published:** 2022-01-26

**Authors:** Peter Paul C. Lim, Lisa M. Stempak, Sindhoosha Malay, LeAnne N. Moore, Sree Sarah S. Cherian, Ankita P. Desai

**Affiliations:** 1 Department of Pediatric Infectious Diseases, University Hospitals-Rainbow Babies and Children’s Hospital, 11100 Euclid Avenue, Cleveland, OH 44106, USA; 2 Department of Pathology, University Hospitals-Cleveland Medical Center, 11100 Euclid Avenue, Cleveland, OH 44106, USA; Lisa.Stempak@UHhospitals.org (L.M.S.); Sree.Cherian@UHhospitals.org (S.S.S.C.); 3 Department of Clinical Research Biostatistics, University Hospitals-Rainbow Babies and Children’s Hospital, 11100 Euclid Avenue, Cleveland, OH 44106, USA; Sindhoosha.Malay@UHhospitals.org; 4 Department of Pediatric Pharmacy, University Hospitals-Rainbow Babies and Children’s Hospital, 11100 Euclid Avenue, Cleveland, OH 44106, USA; LeAnne.Moore@UHhospitals.org

**Keywords:** 16S rRNA sequencing, broad-range PCR, antibiotic stewardship, culture-negative infection, pediatrics

## Abstract

The use of 16S rRNA sequencing in culture-negative infections has improved identification of bacterial pathogens in select scenarios, but its clinical impact requires further elucidation, especially in the pediatric population. This retrospective study aims to determine the clinical utility of 16S rRNA sequencing on the clinical management of pediatric culture-negative infections in our institution. Significant clinical utility was identified in 30 (40.5%) of 74 clinical samples (*p* < 0.0001). Of all specimens, pulmonary samples yielded the most clinical utility (*n* = 9, 30%), followed equally by joint fluid (*n* = 6, 20%) and bone (*n* = 6, 20%), with no difference between fluid and fresh tissue specimens (*p =* 0.346). Although the difference was not statistically significant (*p* = 0.4111), the overall use of broad-spectrum coverage was decreased. The median number of antibiotics was decreased from two to one (*p* < 0.0001) based on 16S rRNA sequencing results. The results suggest that 16S rRNA sequencing has a significant impact on decreasing the number of antibiotics used in the treatment of pediatric culture-negative infections. 16S rRNA sequencing performed on pulmonary specimens has the highest likelihood of identifying a pathogen compared to other specimen types. Additional cost–benefit analysis needs to be completed to further determine clinical benefit.

## 1. Introduction

Isolation and identification of a pathogen in pediatric infections is crucial to facilitate a targeted antimicrobial regimen. This quintessential step is necessary for an optimal outcome in many infectious disease diagnoses. Culture-based methods remain the gold standard to achieve a definitive diagnosis. However, several important limitations cannot be overlooked in the process of attaining directed therapy from bacterial culture identification. Many organisms are fastidious or may be present at low concentrations, and a number of patients may have been empirically treated with broad-spectrum antibiotics before bacterial cultures are collected, often yielding no growth. Thus, the application of molecular diagnostics such as 16S ribosomal RNA (16S rRNA) broad-range polymerase chain reaction (PCR) and next-generation sequencing (NGS) aids in the identification of bacterial pathogens in the aforementioned scenarios and is increasingly utilized as a promising supplemental diagnostic test to achieve tailored antimicrobial management and guided therapy [1,2,3].

16S rRNA sequencing is a broad-range PCR used in identifying bacterial pathogens to the species level by sequencing the highly-conserved bacterial 16S rRNA region [1,2]. Previous studies have demonstrated this molecular application has reasonable concordance when compared to conventional bacterial cultures and has clinical significance [4,5,6]. This technology is especially beneficial, but not limited, to more invasive, difficult-to-diagnose and complicated culture-negative infections where pathogen identification is of high importance. Due to the potential for deleterious effects if these types of infectious processes are mistreated or undertreated, prolonged therapy is often required. With the knowledge of the propensity of the negative effects of broad-spectrum antibiotics, it is critical to mitigate this risk, particularly in children, by employing a more targeted antimicrobial approach [2,3,4,5]. Among the negative outcomes of unnecessary prolonged antimicrobial use is development of antibiotic resistance, which is an urgent major public health threat. The development of *Clostridioides difficile* colitis, drug-related adverse events such as renal toxicity, and even death are other important consequences of unnecessary use and misuse of antibiotics [7,8].

Currently, utilizing 16S rRNA sequencing when conventional cultures do not yield a pathogen is common practice among infectious disease specialists [9]. However, while the laboratory utility of 16S rRNA sequencing has been demonstrated in prior studies, its utility in clinical decision making to guide clinicians on when to order the test and how it influences the selection of antimicrobial management remains to be fully elucidated [2]. Moreover, the majority of the data in relevant literature are extrapolated from studies in adult populations as the use of 16S rRNA sequencing has been increasing, especially in the diagnosis of prosthetic joint infection [10,11]. Several studies have also shown its clinical impact in the management of culture-negative endocarditis [12,13,14,15]. To date, there is a paucity of data on the clinical utility of 16S rRNA sequencing in pediatric infections [2,6]. Few studies highlight the diagnostic utility of 16S rRNA sequencing in select pediatric infections [16,17,18,19,20]. Most of these studies have attempted to isolate its clinical usefulness in the diagnosis of neonatal sepsis [21,22,23,24,25]. However, evidence for its clinical utility and validation of these data in real-world clinical practice in the pediatric population remains limited [26,27,28,29,30]. Perhaps one of the main reasons for its limitation stems from an arbitrary indication of its use in the pediatric population and institutional practice variations, resulting in unclear results reflecting meaningful universal evidence for its routine implementation [2]. The aim of this retrospective cohort study was to evaluate the clinical impact of 16S rRNA sequencing in the management of pediatric bacterial infections in our institution.

## 2. Materials and Methods

### 2.1. Study Design, Setting and Data Collection

The study population consisted of pediatric patients admitted at the University Hospitals-Rainbow Babies and Children’s Hospital (UH-RBC) from August 2016 to March 2020 whose tissue and fluid samples had 16S rRNA sequencing performed. UH-RBC is a 244-bed full-service children’s hospital and academic center located in Cleveland, Ohio. Patients were retrospectively identified by careful manual review of requisition forms, which are mandatory documents serving as proof that the specimen was sent out for 16S rRNA sequencing. 16S rRNA sequencing for all specimens at UH-RBC was performed by the University of Washington Molecular Diagnostics Laboratory (UW). At UW, microbial DNA is isolated from the tissue or fluid specimen and then amplified using conventional PCR utilizing a battery of broad-range primers. The amplified products are then sequenced, which is used to identify the organism based on sequence data [31]. Additional testing details have been published previously [9]. The samples were collected under sterile conditions from operating rooms or in interventional radiology suites. None of the samples included in the study were collected at bedside. The decision to send a clinical specimen was a joint decision between the pediatric infectious disease consultants and the medical directors of the microbiology laboratory. Tissue specimens, including bone specimens, were processed following the microbiology laboratory tissue grinding procedure prior to inoculation on the appropriate media for culture type. Clinical specimens are routinely refrigerated at 2–8 degrees Celsius for seven days in the microbiology laboratory prior to being discarded, which is an institutional protocol. Subsequent surgical procedures were not performed to obtain additional clinical specimens for 16S rRNA sequencing. 16S rRNA sequencing analysis was performed on the remaining portion of the original specimen after conventional cultures were set up. In all cases, 16S rRNA sequencing was performed only after thorough review of conventional culture results after an appropriate incubation period, which is dictated mainly by clinical rationale and discussion between the infectious disease physicians and the directors of the microbiology laboratory. Respiratory specimens and wound cultures are finalized as ‘no growth’ after 36 h of incubation on the BD Kiestra Total Laboratory Automation. Routine sterile fluid cultures (including CSF samples) are finalized as ‘no growth’ after 60 h of incubation on the BD Kiestra Total Laboratory Automation. For select clinical specimen types, extended incubation is performed as deemed clinically necessary.

Basic demographic information such as age and sex, as well as necessary clinical data were extracted from the electronic medical records of patients whose clinical samples were sent for 16S rRNA sequencing. A detailed review of the patient charts was performed to determine the clinical utility of the test results in the patient’s clinical care.

This study was approved by the Institutional Review Board of the University Hospitals-Rainbow Babies and Children’s Hospital.

### 2.2. Definition of Clinical Utility

Clinical utility was defined as a change in a patient’s overall antimicrobial regimen, pathogen confirmation, or impact on treatment duration. A positive result was deemed clinically useful if it confirmed a clinical suspicion of a bacterial pathogen that was compatible with a patient’s clinical condition. Clinically significant antimicrobial regimen changes consisted of narrowing coverage, adding an antimicrobial agent, switching to a different regimen, changing the duration, or discontinuation of therapy based on the 16S rRNA sequencing results. A negative result was considered clinically useful if it confirmed the absence of a suspected pathogen, ruling out the need for broader antimicrobial coverage or discontinuation of a particular antibiotic.

### 2.3. Definition of Broad and Narrow Spectrum Coverage

Broad-spectrum coverage was defined as any regimen containing vancomycin, cefepime, piperacillin-tazobactam or meropenem, or any empiric regimen whose coverage was never changed from initiation of therapy. Narrow-spectrum coverage is defined as any de-escalation of any empiric regimen to a more targeted therapy based on 16S rRNA sequencing results.

### 2.4. Statistical Analysis

Clinical characteristics of the patients were described using median and interquartile range (25th percentile, 75th percentile) for continuous variables and frequency and percentages for categorical variables as appropriate. Categorical variables were analyzed using the Fisher exact test. Continuous variables were assessed by the Wilcoxon rank sum test. Paired analyses were carried out by paired t-test and McNemar’s test. A *p*-value of less than 0.05 was considered statistically significant for the analysis. All of the analyses were performed using SAS software, version 9.4 (SAS Institute, Cary, NC, USA) and R software, version 4.0.4.

## 3. Results

**Figure 1:**
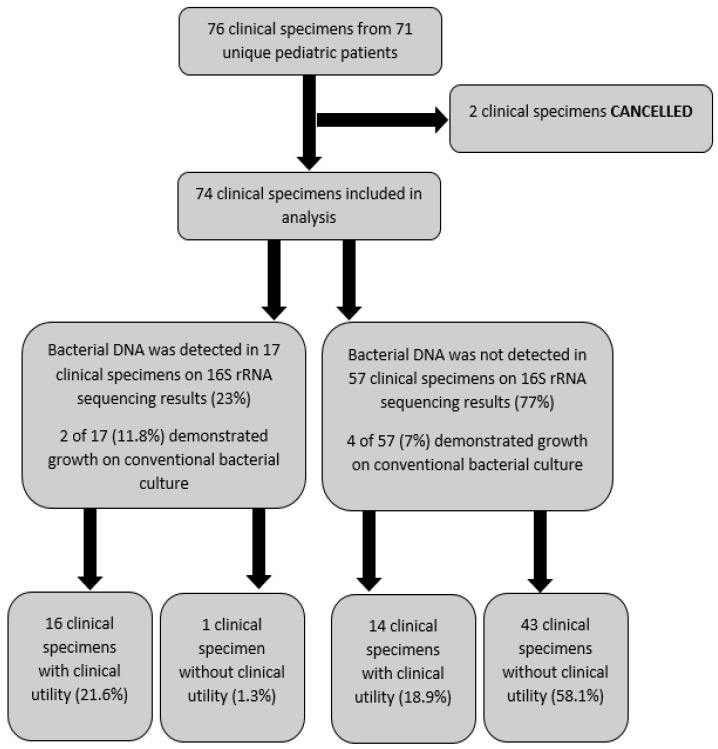
Diagram demonstrating the clinical utility outcome for the pediatric specimens that had 16S rRNA sequencing performed in UH-RBC from August 2016 to March 2020.

A total of 76 specimens from 71 unique patients at UH-RBC had 16S rRNA sequencing performed during the study period. Two of the specimens were excluded because they were cancelled for reasons that could not be extrapolated from detailed chart review. A total of 74 samples were included in the final analysis. Thirty-two (45%) patients were male and 39 (55%) were female, with a median age of 8 (IQR 4,13) (Figure 1).

Seventeen (23%) of these seventy-four samples demonstrated a pathogen on 16S rRNA sequencing results, and 57 (77%) did not detect a pathogen on 16S rRNA sequencing results (Table 1 and Table S1). The most common organism that 16S rRNA sequencing identified was *Streptococcus pneumoniae* (*n* = 5, 29.4%). This was followed equally by *Kingella kingae* (*n* = 3, 17.6%) and collectively by other *Streptococcus* species (*Streptococcus intermedius, Streptococcus pyogenes, Streptococcus sanguinis)* from different clinical specimens (Table 1).

Of the 74 specimens that had 16S rRNA testing performed, 32 (43%) were fluid specimens and 42 (57%) were tissue specimens. The most common specimen sent for 16S rRNA sequencing was joint fluid (*n* = 18, 24%), followed by pulmonary samples (*n* = 15, 20%) and bone samples (*n* = 14, 19%). Pulmonary samples comprised lung tissue specimens, bronchoalveolar lavage (BAL) washings and pleural fluid specimens.

**Figure 2:**
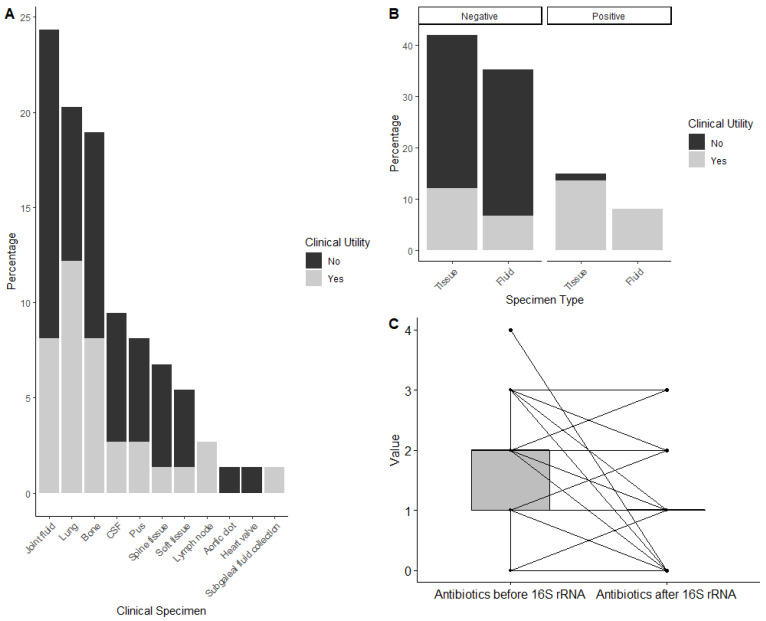
(**A**) The clinical utility of 16S rRNA sequencing results by clinical specimen type. (**B**) Comparison of clinical utility of 16S rRNA sequencing results between tissue and fluid samples (**C**) Paired visualization of the change in the number of antibiotics impacted by 16S rRNA sequencing results. (Legend: Figure 2B ‘Negative’ = No bacterial DNA detected; ‘Positive’ = With bacterial DNA detected.)

Significant clinical utility was identified in 30 (40.5%) of 74 clinical specimens (*p* < 0.0001) (Table 1). A supplemental table can be found in the supplemental material detailing clinical specimens that did not show clinical utility (Table S1). Of all specimens demonstrating clinical significance, pulmonary samples yielded the most clinical utility (*n* = 9, 30%), followed equally by joint fluid (*n* = 6, 20%) and bone (*n* = 6, 20%). There was no significant difference in clinical utility between fluid and tissue specimens (*p =* 0.346) (Figure 2A,B).

The most common clinical diagnosis found to warrant 16S rRNA sequencing was osteomyelitis (*n* = 24, 32%), followed by abscess (*n* = 13, 18%), pneumonia (*n* = 12, 16%) and septic arthritis (*n* = 11, 15%). There are significant differences among the spectrum of clinical diagnoses found to have clinical utility based on 16S rRNA sequencing results (*p* = 0.021).

The standard protocol for UH-RBC is to perform 16S rRNA sequencing when conventional bacterial culture shows no growth after the standard incubation period. However, 6 (8%) of the 74 clinical specimens that had 16S rRNA sequencing performed eventually yielded growth on conventional culture (Figure 1). Two specimens that yielded positive 16S rRNA results had conventional bacterial culture growth that corresponded to the pathogens identified by 16S rRNA sequencing demonstrating clinical utility. One patient grew *Streptococcus intermedius* from an orbital subperiosteal abscess, and another patient grew *Prevotella nanceiensis* from subgaleal fluid collection after 2 and 5 days of incubation, respectively. Four patients with negative 16S rRNA sequencing results subsequently exhibited growth with pathogen identification by conventional bacterial culture. Two of these patients had negative 16S rRNA sequencing results from cerebrospinal fluid (CSF) and spinal tissue samples but eventually grew non-typeable *Haemophilus influenzae* and *Streptococcus agalactiae* on blood culture, respectively. One other patient did not demonstrate a pathogen on 16S rRNA sequencing results from a thigh abscess specimen but eventually grew *Pseudomonas aeruginosa* on standard bacterial culture after 4 days of incubation. Another patient with no pathogen detected by 16S rRNA sequencing from a trapezius muscle specimen demonstrated growth of *Escherichia vulneris* on conventional bacterial culture after 1 day of incubation. All growth in the conventional cultures was observed after the 16S rRNA testing had been sent to the reference laboratory for testing.

Out of the 71 total patients, 64 patients warranted antibiotics both pre- and post-16S rRNA sequencing result. The other seven patients either were not empirically started on any antibiotics, did not require antibiotics throughout their course as other evolving clinical parameters did not suggest an infectious etiology, or the antibiotic regimen was discontinued before the 16S rRNA sequencing was completed. In the 64 patients whose antimicrobial spectrum coverage was analyzed, patients requiring broad-spectrum coverage decreased from 48 to 21 (75% to 33%) and narrow-spectrum coverage increased from 16 to 43 (25% to 67%) based on the 16S rRNA sequencing results, though neither was statistically significant (*p* = 0.4111). Of all patients included in the analysis, the median number of antibiotics used before the 16S rRNA sequencing was completed significantly decreased from 2 to 1 (*p* < 0.0001) (Figure 2C).

## 4. Discussion

Timely identification of an infectious pathogen is of utmost importance to formulate a targeted antimicrobial regimen [2,3,4,5,8]. This is especially imperative in more serious or invasive infections that warrant prolonged treatment where a more focused therapy can mitigate the risks of adverse reactions, especially in pediatric patients. This study aimed to evaluate the clinical impact of 16S rRNA sequencing in the management of pediatric infections. We found that 16S rRNA sequencing resulted in significant clinical utility in 40.5% of our clinical specimens, defined by an overall change in antimicrobial management and clinical decision-making. 16S rRNA sequencing has also shown to have a significant impact on decreasing the number of antibiotics used in the management of these infections.

This study is one of the few to assess the clinical utility of 16S rRNA sequencing in pediatric patients across different clinical specimens. Prior studies in children have limited focus on the use of 16S rRNA sequencing in select clinical specimens. Identification of fastidious organisms, specifically *Kingella kingae*, through targeted PCR in primary septic arthritis is one application that has been shown to be clinically useful in children [16,17,18]. One of these studies has particularly shown that targeted PCR is a useful adjunct to diagnostic and treatment modalities, providing important supplemental information compared to what is provided solely by standard bacterial culture results [18]. Attempts to determine the use of this molecular tool in the diagnosis of meningitis has also been made, although the clinical relevance remains ambiguous due to very small sample sizes in these studies [19,29]. In the same way, other researchers have looked into its clinical importance in blood and urine samples in the diagnosis of neonatal sepsis [20,21,22,23,24,25,26,27,28]. Most of these studies have demonstrated important clinical worth of 16S rRNA sequencing in the diagnosis of particular infections in children, albeit there are differences in the diagnostic yield across these studies brought about by factors such as specimen types and differing institutional practices.

Most of the aforementioned studies have compared the 16S rRNA sequencing results with conventional culture results as the gold standard and extrapolated the clinical usefulness of these methods from this comparison. Several other studies in the adult population have shown considerable clinical value of 16S rRNA sequencing across select clinical specimens, although its value in clinical decision making remains hypothetical in lieu of further larger validation studies [5,8,9,10,11,12,13,14,21]. In this study, we exclusively reviewed cases from pediatric patients whose conventional cultures were negative at the time 16S rRNA sequencing was performed. The definition of clinical utility in this study was based on multi-faceted clinical information derived from detailed chart review and not just on the sole basis of detection of pathogens in 16S rRNA sequencing results. Some 16S rRNA sequencing results that did not yield a pathogen have been determined to be clinically useful on the basis of their role in making changes in the overall clinical management of the patient.

One particular study, although performed in adult patients, used a similar approach where the researchers evaluated the clinical usefulness of universal broad-range PCR with other laboratory results using a “composite clinical diagnosis”. This particular study found there was an alteration in management in 18 patients (11 with positive universal PCR results, 7 with negative universal PCR results) [8]. A similar, larger retrospective study using 1062 clinical specimens in 864 combined adult and pediatric patients (those less than 18 years old accounted for 15.5% of all included) found clinically significant results in 107 of 1062 samples (10.1%) that resulted in clinical management change in 44 of 1062 samples (4.1%) when real-world utility of 16S rRNA sequencing was used in pathogen detection [2]. Our results, on the other hand, showed clinical utility in 40.5% of clinical specimens, which is a significantly higher number. This may be explained by the more stringent definition of clinical utility in the study performed by Kerkhoff et al. and by the fact that their pediatric sample representation was only 15.5% of the total patient population, compared to ours which was represented exclusively by pediatric patients. Unfortunately, the isolated clinical utility specifically for pediatric patients included in their study was not elucidated in the paper. In addition, the impact of negative universal broad-range PCR results on the patient’s clinical management in this particular study was not evaluated, which could have further decreased the proportion of specimens that could have resulted in clinical usefulness.

Our study has shown that there is no significant difference in terms of clinical utility of 16S rRNA sequencing between fluid and tissue samples. This is in contrast to a prior, similar study that was performed in a mixture of adult and pediatric patients, which showed that tissue-based specimens are more likely to demonstrate clinically significant results [2]. It is hard to delineate a particular reason for this difference when the results of this study with a mixed population did not show separated analysis between adult and pediatric patients. This may be due to the discrepancy in sample types brought about by differences in disease process epidemiology between adult and pediatric patients, causing more types of samples to be obtained for a particular disease process than another. Another reason would have been differences in pre-analytic factors, such as differences in the volume of specimens obtained between adult and pediatric patients.

One unique finding of our study is the determination of pulmonary specimens as the clinical specimen that yielded the most clinical significance among all clinical specimen types tested. This was followed equally by joint fluid and bone samples. The most likely reason for this is the larger subpopulation of patients with pulmonary clinical diagnosis in our cohort compared to other diagnoses. This correlated to the results of a prior study that showed pulmonary specimens, which consisted of pleural fluid, lung tissue and BAL, yielded the most clinical utility of all specimens. In this particular study, the next best sample type that yielded clinical usefulness was bone specimens followed by joint fluid [2].

The main strength of our study is its focus on determining the clinical value of 16S rRNA sequencing, specifically in pediatric infections across a wide variation of clinical samples. The approach included a detailed chart review to determine the role of 16S rRNA sequencing based on individualized decisions documented in patient charts, which, although bias-prone, showed the comprehensive value of this tool in the specific change it effected in the management of the patient’s overall clinical plan. Moreover, this study also demonstrated some additional data about which clinical sample type is more likely to yield relevant clinical value over another, which is an important question that needs clear elucidation to help guide clinicians to maximize the use of this diagnostic tool.

There are some important limitations worth acknowledging when interpreting the results of our study. First, the small sample size could have limited the statistical power of our study. Second, it was conducted at a single institution. Third, it is a retrospective study using chart review to determine clinical utility of 16S rRNA sequencing in managing pediatric infections which could be misclassified, although this chance is lessened given the chart review which documented how the 16S rRNA sequencing results changed the overall clinical plans. Fourth, clinical outcomes were not evaluated beyond proximal decisions around antibiotic regimen changes and immediate clinical plans for the patients following 16S rRNA sequencing results. Fifth, there is always a possibility that other unmeasured factors we did not evaluate in this study could have influenced the clinical decision changes of the providers, which may be outside what is documented in the charts. It is also important to acknowledge that we only included samples with culture-negative results in this study. The results of this study should be carefully interpreted outside this clinical context. It is also important to mention some of the limitations of this molecular method that may result from sampling, storing and processing techniques that may lead to contamination of samples and falsely show erroneous results. This calls for prudent clinical judgment to be taken into account when interpreting the results.

## 5. Conclusions

In conclusion, 16S rRNA sequencing has a significant impact in terms of decreasing the number of antibiotics used in the treatment of pediatric infections. It is also a potentially useful clinical tool to establish a diagnosis in culture-negative infections. The performance of 16S rRNA sequencing in demonstrating clinical utility differs across clinical specimens but did not show a significant difference between tissue and fluid samples. Pulmonary specimens have the highest clinical utility among all samples. Additional cost–benefit analysis needs to be completed to further determine clinical benefit.

## Figures and Tables

**Table 1:** Summary of specimen types, conventional culture and 16S rRNA sequencing results, empiric antibiotic regimen and post-16S rRNA sequencing result antibiotic changes, and overall clinical impact of the 16S rRNA sequencing results to the clinical decision-making process of the 16S rRNA clinical samples that demonstrated clinical utility. (Abbreviations: TMP-SMX = Trimethroprim-Sulfamethoxazole).

Patient	Specimen Type	Antimicrobial Regimen before 16S rRNA Sequencing Result	Conventional Culture Result	16S rRNA Sequencing Result	Antimicrobial Regimen after 16S rRNA Sequencing Result	Clinical Diagnosis	Clinical Impact
1	Bronchoalveolar lavage	Amphotericin/Meropenem/ Linezolid	No growth	*Candida parapsilosis*	Amphotericin/Meropenem/Linezolid	Candidal pneumonia	Confirmed an organism
2	Bone	Vancomycin/Cefepime/Metronidazole	No growth	No bacterial DNA detected	Ampicillin-sulbactam	Left paraspinal abscess	Ruled out Methicillin Resistant *Staphylococcus aureus*
3	Bone	Vancomycin/Ceftriaxone	No growth	*Corynebacterium tuberculostearicum*	Ceftriaxone/TMP-SMX/Azithromycin	Multifocal osteomyelitis, septic arthritis	Confirmed an organism
4	Bone	Doxycyline/Ciprofloxacin	No growth	No bacterial DNA detected	Ciprofloxacin	Right Chronic trapezoid osteomyelitis	Ruled out suspected organism
5	Bone-Right Femur	Vancomycin/Ceftriaxone	No growth	No bacterial DNA detected	Amoxicillin	Lyme arthritis/Right knee osteomyelitis	Ruled out Staphylococcal infection
6	Bone-Mastoid	Vancomycin/Piperacillin-tazobactam	No growth	*Streptococcus pneumoniae*	Ceftriaxone	Mastoid abscess	Narrowed down antibiotic coverage
7	Bone-Vertebral body/spinal biopsy	None	No growth	*Kingella kingae*	Ceftriaxone	Vertebral osteomyelitis	Confirmed an organism
8	Cerebrospinal fluid	Vancomycin/Ceftriaxone	No growth	No bacterial DNA detected	None	Aseptic meningitis	Ruled out an infectious process
9	Cerebrospinal fluid	Nafcillin/Gentamicin/Cefepime	No growth	No bacterial DNA detected	Cefepime	Pseudomonas bacteremia	Ruled out concomitant central nervous system infection
10	Joint fluid	Linezolid	No growth	*Streptococcus sanquinis*	Linezolid	R hip septic arthritis	Confirmed an organism
11	Joint fluid-Elbow aspirate	Clindamycin	No growth	*Kingella kingae*	Amoxicillin-clavulanic acid	Left elbow septic arthritis, osteomyelitis	Confirmed an organism
12	Joint fluid-Hip	None	No growth	No bacterial DNA detected	None	Bilateral hip effusion	Ruled out infectious process
13	Joint fluid-Hip aspirate	Cefazolin	No growth	*Kingella kingae*	Amoxicillin-clavulanic acid	Right hip septic arthritis	Confirmed an organism
14	Joint fluid-Hip fluid	Cefazolin	No growth	*Propionibacterium acnes*	Cephalexin	Left hip septic arthritis	Confirmed an organism
15	Joint fluid-Left elbow	Vancomycin/Ceftriaxone	No growth	No bacterial DNA detected	Cephalexin	Left elbow chronic osteomyelitis	Ruled out suspected resistant Gram-positive organism
16	Lung biopsy	N/A	No growth	No bacterial DNA detected	None	Lung nodule	Ruled out infectious process
17	Lymph node	Azithromycin/Rifampin	No growth	*Bartonella* species	None	Bartonella lymphadenitis	Confirmed an organism
18	Lymph node	Azithromycin/Ethambutol/Levofloxacin	No growth	No bacterial DNA detected	None	Reactive lymphadenitis	Ruled out an infectious process
19	Pleural fluid	Vancomycin/Clindamycin/Ceftriaxone	No growth	*Streptococcus pneumoniae*	Amoxicillin-clavulanic acid	Complicated pneumonia	Confirmed an organism
20	Pleural fluid	Ceftriaxone/Vancomycin	No growth	*Streptococcus pneumoniae*	Ceftriaxone	Complicated pneumonia	Narrowed down antibiotic coverage
21	Pleural fluid	Piperacillin-tazobactam	No growth	No bacterial DNA detected	Piperacillin-tazobactam	Intraabdominal infection/pleural effusion	Ruled out concomitant lung infection
22	Pleural fluid	Ceftriaxone	No growth	No bacterial DNA detected	None	Pleural effusion	Ruled out infectious cause
23	Pleural fluid	Vancomycin/Ceftriaxone	No growth	*Streptococcus pneumoniae*	Ceftriaxone/Clindamycin	Complicated pneumonia	Confirmed an organism
24	Pleural fluid	Vancomycin/Ceftriaxone/Azithromycin	No growth	*Streptococcus pyogenes*	Amoxicillin-clavulanic acid	Complicated pneumonia	Confirmed an organism
25	Pleural fluid	Ceftriaxone/Vancomycin/Linezolid	No growth	*Streptococcus pneumoniae*	Ampicillin	Lung abscess	Confirmed an organism
26	Pus-Pustule fluid	Piperacillin-tazobactam->Ampicillin-sulbactam->Amoxicillin-clavulanic acid	No growth	No bacterial DNA detected	Amoxicillin-clavulanic acid	Right cheek skin infection/drainage	Ruled out Non-Mycobacterial Tuberculosis
27	Pus-Subperiosteal abscess	Vancomycin/Ceftriaxone	*Streptococcus intermedius*	*Streptococcus intermedius*	Amoxicillin-clavulanic acid	Left orbital cellulitis/subperiosteal abscess	Confirmed an organism
28	Soft tissue-Neck mass	Amoxicillin-clavulanic acid	No growth	No bacterial DNA detected	Amoxicillin-clavulanic acid	Cervical ymphadenitis	Ruled out Bartonella and Non-Mycobacterial tuberculosis
29	Spine tissue-Deep spine tissue	Vancomycin/Cefepime/Metronidazole	No growth	No bacterial DNA detected	Ampicillin-sulbactam	Left paraspinal abscess	Ruled out Methicillin Resistant *Staphylococcus aureus*
30	Subgaleal fluid collection	Cefepime/Vancomycin	*Prevotella* sp., *Candida lusitaniae*	*Prevotella nanceiensis*	Meropenem	Subgaleal abscess	Confirmed an organism

## Data Availability

Not applicable.

## Supplementary Materials

The following supporting information can be downloaded at: https://www.mdpi.com/article/10.3390/antibiotics11020159/s1. Table S1: Summary of specimen types, conventional culture and 16S rRNA sequencing results, empiric antibiotic regimen and post-16S rRNA sequencing result antibiotic changes and overall clinical impact of the 16S rRNA sequencing result to the clinical decision making process of the 16S rRNA clinical samples that demonstrated no clinical utility. (Abbreviations: TMP-SMX = Trimethroprim-Sulfamethoxazole).

## References

[1] Patel A., Harris K.A., Fitzgerald F. (2017). What is broad-range 16S rDNA PCR?. Arch. Dis. Child.-Educ. Pract..

[2] Kerkhoff A.D., Rutishauser R.L., Miller S., Babik J.M. (2020). Clinical Utility of Universal Broad-Range Polymerase Chain Reaction Amplicon Sequencing for Pathogen Identification: A Retrospective Cohort Study. Clin. Infect. Dis..

[3] Drancourt M., Bollet C., Carlioz A., Martelin R., Gayral J.P., Raoult D. (2000). 16S ribosomal DNA sequence analysis of a large collection of environmental and clinical unidentifiable bacterial isolates. J. Clin. Microbiol..

[4] Akram A., Maley M., Gosbell I., Nguyen T., Chavada R. (2017). Utility of 16S rRNA PCR performed on clinical specimens in patient management. Int. J. Infect. Dis..

[5] Rampini S.K., Bloemberg G.V., Keller P.M., Büchler A.C., Dollenmaier G., Speck R.F., Böttger E.C. (2011). Broad-range 16S rRNA gene polymerase chain reaction for diagnosis of culture-negative bacterial infections. Clin. Infect. Dis..

[6] Fida M., Khalil S., Abu Saleh O., Challener D.W., Sohail M.R., Yang J.N., Pritt B.S., Schuetz A.N., Patel R. (2021). Diagnostic value of 16S ribosomal RNA gene polymerase chain reaction/Sanger sequencing in clinical practice. Clin. Infect. Dis..

[7] Nelson R.E., Hatfield K.M., Wolford H., Samore M.H., Scott R.D., Reddy S.C., Olubajo B., Paul P., Jernigan J.A., Baggs J. (2021). National estimates of healthcare costs associated with multidrug-resistant bacterial infections among hospitalized patients in the United States. Clin. Infect. Dis..

[8] Kadri S.S. (2020). Key takeaways from the US CDC’s 2019 antibiotic resistance threats report for frontline providers. Crit. Care Med..

[9] Basein T., Gardiner B.J., Andujar Vazquez G.M., Joel Chandranesan A.S., Rabson A.R., Doron S., Snydman D.R. (2018). Microbial identification using DNA target amplification and sequencing: Clinical utility and impact on patient management. Open Forum Infect. Dis..

[10] Bémer P., Plouzeau C., Tande D., Léger J., Giraudeau B., Valentin A.S., Jolivet-Gougeon A., Vincent P., Corvec S., Gibaud S. (2014). Evaluation of 16S rRNA gene PCR sensitivity and specificity for diagnosis of prosthetic joint infection: A prospective multicenter cross-sectional study. J. Clin. Microbiol..

[11] Kobayashi N., Procop G.W., Krebs V., Kobayashi H., Bauer T.W. (2008). Molecular identification of bacteria from aseptically loose implants. Clin. Orthop. Relat. Res..

[12] Marín M., Muñoz P., Sánchez M., del Rosal M., Alcalá L., Rodríguez-Créixems M., Bouza E., Group for the Management of Infective Endocarditis of the Gregorio Marañón Hospital (GAME) (2007). Molecular diagnosis of infective endocarditis by real-time broad-range polymerase chain reaction (PCR) and sequencing directly from heart valve tissue. Medicine.

[13] Fournier P.E., Thuny F., Richet H., Lepidi H., Casalta J.P., Arzouni J.P., Maurin M., Célard M., Mainardi J.-L., Caus T. (2010). Comprehensive diagnostic strategy for blood culture-negative endocarditis: A prospective study of 819 new cases. Clin. Infect. Dis..

[14] Faraji R., Behjati-Ardakani M., Moshtaghioun S.M., Kalantar S.M., Namayandeh S.M., Soltani M., Emami M., Zandi H., Firoozabadi A.D., Sarebanhassanabadi M. (2018). The diagnosis of microorganism involved in infective endocarditis (IE) by polymerase chain reaction (PCR) and real-time PCR: A systematic review. Kaohsiung J. Med. Sci..

[15] Bosshard P.P., Kronenberg A., Zbinden R., Ruef C., Böttger E.C., Altwegg M. (2003). Etiologic diagnosis of infective endocarditis by broad-range polymerase chain reaction: A 3-year experience. Clin. Infect. Dis..

[16] Villani M.C., Hamilton E.C., Klosterman M.M., Jo C., Kang L.H., Copley L.A. (2021). Primary septic arthritis among children 6 to 48 months of age: Implications for PCR acquisition and empiric antimicrobial selection. J. Pediatric Orthop..

[17] Slinger R., Moldovan I., Bowes J., Chan F. (2016). Polymerase chain reaction detection of Kingella kingae in children with culture-negative septic arthritis in eastern Ontario. Paediatr. Child Health.

[18] Carter K., Doern C., Jo C.H., Copley L.A. (2016). The clinical usefulness of polymerase chain reaction as a supplemental diagnostic tool in the evaluation and the treatment of children with septic arthritis. J. Pediatric Orthop..

[19] Abelian A., Mund T., Curran M.D., Savill S.A., Mitra N., Charan C., Ogilvy-Stuart A.L., Pelham H.R.B., Dear P.H. (2020). Towards accurate exclusion of neonatal bacterial meningitis: A feasibility study of a novel 16S rDNA PCR assay. BMC Infect. Dis..

[20] İstanbullu K., Köksal N., Çetinkaya M., Özkan H., Yakut T., Karkucak M., Doğan H. (2019). The potential utility of real-time PCR of the 16S-rRNA gene in the diagnosis of neonatal sepsis. Turk. J. Pediatrics.

[21] Stranieri I., Kanunfre K.A., Rodrigues J.C., Yamamoto L., Nadaf MI V., Palmeira P., Okay T.S. (2018). Assessment and comparison of bacterial load levels determined by quantitative amplifications in blood culture-positive and negative neonatal sepsis. Rev. Do Inst. De Med. Trop. De São Paulo.

[22] Punia H., Gathwala G., Dhaulakhandi D.B., Aamir M. (2017). Diagnosis of neonatal sepsis using 16S rRNA polymerase chain reaction. Trop. Dr..

[23] Midan D.A., Abo El Fotoh WM M., El Shalakany A.H. (2017). The potential role of incorporating real-time PCR and DNA sequencing for amplification and detection of 16S rRNA gene signatures in neonatal sepsis. J. Matern.-Fetal Neonatal Med..

[24] Stranieri I., Kanunfre K.A., Rodrigues J.C., Yamamoto L., Nadaf MI V., Palmeira P., Okay T.S. (2016). Usefulness of a 16S rDNA real-time PCR to monitor neonatal sepsis and to assist in medical decision to discontinue antibiotics. J. Matern.-Fetal Neonatal Med..

[25] Das B.K., Suri S., Nath G., Prasad R. (2015). Urine nested polymerase chain reaction in neonatal septicemia. J. Trop. Pediatrics.

[26] Al-Zahrani A.K., Ghonaim M.M., Hussein Y.M., Eed E.M., Khalifa A.S., Dorgham L.S. (2015). Evaluation of recent methods versus conventional methods for diagnosis of early-onset neonatal sepsis. J. Infect. Dev. Ctries..

[27] Liu C.L., Ai H.W., Wang W.P., Chen L., Hu H.B., Ye T., Zhu X., Wang F., Liao Y., Wang Y. (2014). Comparison of 16S rRNA gene PCR and blood culture for diagnosis of neonatal sepsis. Arch. De Pédiatrie.

[28] Ohlin A., Bäckman A., Ewald U., Schollin J., Björkqvist M. (2012). Diagnosis of neonatal sepsis by broad-range 16S real-time polymerase chain reaction. Neonatology.

[29] Esparcia O., Montemayor M., Ginovart G., Pomar V., Soriano G., Pericas R., Gurgui M., Sulleiro E., Prats G., Navarro F. (2011). Diagnostic accuracy of a 16S ribosomal DNA gene-based molecular technique (RT-PCR, microarray, and sequencing) for bacterial meningitis, early-onset neonatal sepsis, and spontaneous bacterial peritonitis. Diagn. Microbiol. Infect. Dis..

[30] Ammann R.A., Zucol F., Aebi C., Niggli F.K., Kühne T., Nadal D. (2007). Real-time broad-range PCR versus blood culture. A prospective pilot study in pediatric cancer patients with fever and neutropenia. Supportive Care Cancer.

[31] University of Washington Department of Laboratory Medicine: Molecular Diagnosis Microbiology Section. http://depts.washington.edu/molmicdx/mdx/available_tests.shtml.

