# Supplementary material for: Determining the Clinical Utility of 16S rRNA Sequencing in the Management of Culture-Negative Pediatric Infections
Source: Antibiotics (Basel). 2022 Jan 26;11(2):159. doi: 10.3390/antibiotics11020159 (PMC8868208; doi:10.3390/antibiotics11020159)
Supplement: Supplementary file 1 [file antibiotics-11-00159-s001.zip › antibiotics-1545259-supplementary.pdf]

Supplemental Table S1. Summary of specimen types, conventional culture and 16S rRNA sequencing results, empiric antibiotic regimen and post-16S rRNA sequencing result antibiotic changes and overall clinical impact of the 16S rRNA sequencing result to the clinical decision making process of the 16S rRNA clinical samples that demonstrated no clinical utility. (Abbreviations: TMP-SMX=Trimethroprim-Sulfamethoxazole)

Patient	Specimen Type	Antimicrobial Regimen Before 16S rRNA Sequencing Result	Conventional Culture Result	16S rRNA Sequencing Result	Antimicrobial Regimen After 16S rRNA Sequencing Result	Clinical Diagnosis	Clinical Impact of 16S rRNA Sequencing Result
31	Aortic clot	Vancomycin/Gentamicin/Ceftriaxone	No growth	Multiple bacterial DNA templates detected	Ceftriaxone	Mycotic aneurysm	No clinical utility
32	Bronchoalveolar lavage	Cefepime/Vancomycin	No growth	No bacterial DNA detected	Vancomycin/Meropenem	Multifocal pneumonia	No clinical utility
33	Bone	Cefazolin	No growth	No bacterial DNA detected	Cephalexin	Left hip chronic osteomyelitis	No clinical utility
34	Bone - Femur biopsy/tissue	Cefazolin/Clindamycin	No growth	No bacterial DNA detected	Cephalexin	Left femur osteomyelitis	No clinical utility
35	Bone - Iliac bone	Ceftriaxone/Clindamycin	No growth	No bacterial DNA detected	Ceftriaxone/Linezolid	Left hip osteomyelitis	No clinical utility
36	Bone - Left femur	Cefazolin	No growth	No bacterial DNA detected	Cephalexin	Right tibia osteomyelitis and subperiosteal abscess	No clinical utility
37	Bone - Periosteum	Cefazolin	No growth	No bacterial DNA detected	Cephalexin	Left tibial osteonyelitis/Brodie's abscess	No clinical utility
38	Bone biopsy - Right first toe	Clindamycin	No growth	No bacterial DNA detected	Clindamycin	Right great toe chronic osteomyelitis	No clinical utility
39	Bone - calcaneus	Cefepime/Clindamycin	No growth	No bacterial DNA detected	Cefepime/Clindamycin	Right calcaneal chronic osteomyelitis	No clinical utility
40	Bone infarct - Left femur	Ceftriaxone	No growth	No bacterial DNA detected	Amoxicillin-clavulanic acid	Left distal femur osteomyelitis	No clinical utility
41	Cerebrospinal fluid	Vancomycin/Ceftriaxone	No growth	No bacterial DNA detected	Linezolid/Ceftriaxone	Lumbar site wound infection/presumed meningitis	No clinical utility
42	Cerebrospinal fluid	Vancomycin/Cefepime/Gentamicin/Acyclovir	No growth	No bacterial DNA detected	None	Viral rhombencephalitis	No clinical utility
43	Cerebrospinal fluid	Cefepime/Vancomycin	No growth	No bacterial DNA detected	Cefepime/Vancomycin	Presumed Ventriculo-peritoneal shunt infection	No clinical utility
44	Cerebrospinal fluid	Cefepime/Vancomycin	No growth	No bacterial DNA detected	Cefepime/Linezolid	Presumed meningitis	No clinical utility
45	Cerebrospinal fluid	Piperacillin-tazobactam/Gentamicin	No growth but nontyepable *Haemophilus influenzae* in blood culture	No bacterial DNA detected	Meropenem/Gentamicin	Non-typeable *Haemophilus influenzae* bacteremia/ meningitis	No clinical utility

46	Heart valve (melody and conduit)	Vancomycin/Rifampin/Doxycycline	No growth but Bartonella titers >1:1024 in serum	No bacterial DNA detected	Doxycycline	Infective endocarditis	No clinical utility
47	Joint fluid	Vancomycin/Ceftriaxone	No growth	No bacterial DNA detected	Ceftriaxone	Septic arthritis	No clinical utility
48	Joint fluid - Elbow fluid	Cefazolin/Clindamycin	No growth	No bacterial DNA detected	Cefadroxyl	Left humeral chronic osteomyelitis	No clinical utility
49	Joint fluid - Hip aspirate	Vancomycin/Ceftriaxone	No growth	No bacterial DNA detected	Cephalexin	Right hip septic arthritis	No clinical utility
50	Joint fluid - Hip fluid	Vancomycin/Ceftriaxone	No growth	No bacterial DNA detected	Clindamycin	Left hip septic arthritis	No clinical utility
51	Joint fluid - Left hip joint fluid	Cefepime	No growth	No bacterial DNA detected	TMP-SMX	Left hip septic arthritis, osteomyelitis, urinary tract infection	No clinical utility
52	Joint fluid - Right hip fluid	Vancomycin/Ceftriaxone	No growth	No bacterial DNA detected	Cephalexin	Right hip septic arthritis and osteomyelitis	No clinical utility
53	Joint fluid - Synovial fluid	Vancomycin/Ceftriaxone	No growth	No bacterial DNA detected	Doxycycline	Left septic elbow	No clinical utility
54	Joint fluid - Synovial fluid	Cefazolin	No growth	No bacterial DNA detected	Cephalexin	Left knee septic arthritis	No clinical utility
55	Joint fluid - Synovial fluid	Cefazolin	No growth	No bacterial DNA detected	Cephalexin	Left elbow chronic osteomyelitis	No clinical utility
56	Joint fluid - Synovial fluid	Vancomycin/Ceftriaxone	No growth	No bacterial DNA detected	Cefuroxime	Right knee septic arthritis	No clinical utility
57	Joint fluid - Synovial fluid	Cefazolin	No growth	No bacterial DNA detected	Cefazolin	Left elbow septic arthritis and osteomyelitis	No clinical utility
58	Joint fluid - Synovial fluid	Cefazolin	No growth	No bacterial DNA detected	Cephalexin	Right hip septic arthritis	No clinical utility
59	Lung tissue	Vancomycin/Meropenem/Posaconazole	No growth	No bacterial DNA detected	Cefepime	Multifocal pneumonia	No clinical utility
60	Pleural fluid	None	No growth	No bacterial DNA detected	None	Fever of unknown origin	No clinical utility
61	Pleural fluid	Vancomycin/Ceftriaxone	No growth	No bacterial DNA detected	Amoxicillin-clavulanic acid/TMP-SMX	Empyema	No clinical utility
62	Pleural fluid	Ceftriaxone/Azithromycin	No growth	No bacterial DNA detected	Amoxicillin-clavulanic acid	Complicated pneumonia	No clinical utility
63	Pleural fluid	Levofloxacin	No growth	No bacterial DNA detected	None	Left Lower Lobe pneumonia with pleural effusion	No clinical utility
64	Pus - Abscess	Vancomycin/Meropenem	No growth	No bacterial DNA detected	Meropenem	Spinal osteo, hardware infection	No clinical utility
65	Pus - Intraabdominal abscess	Ciprofloxacin/Metronidazole	No growth	No bacterial DNA detected	Aztreonam/Metronidazole	Recurrent intraabdominal abscess	No clinical utility
66	Pus - Thigh abscess	Cefazolin	*Pseudomonas aeruginosa* on culture	No bacterial DNA detected	Ciprofloxacin	Right thigh abscess	No clinical utility

67	Pus - Wound stump/draining sinus	Vancomycin/Cefepime	No growth	No bacterial DNA detected	TMP-SMX	Below the Knee Amputation Stump Ostomyelitis	No clinical utility
68	Soft tissue - Biopsy back	Vancomycin/Cefepime	No growth	No bacterial DNA detected	Ceftriaxone/Linezolid	Spinal hardware infection	No clinical utility
69	Soft tissue - Left thigh tissue	TMP-SMX/Meropenem/Linezolid	No growth	No bacterial DNA detected	Meropenem/Linezolid	Abscess thigh and arm	No clinical utility
70	Soft tissue - Tissue	Piperacillin-tazobactam	*Escherichia vulneris* on culture	No bacterial DNA detected	Doxycycline	R Chronic trapezoid osteomyelitis	No clinical utility
71	Spine tissue (Lumbar 1-Lumbar 2 disk)	Cefazolin	No growth	No bacterial DNA detected	Cephalexin	L1/L2 disciitis with osteomyelitis	No clinical utility
72	Spine tissue - Perispinal abscess hardware/tissue	Vancomycin/Cefepime	No growth	No bacterial DNA detected	Ceftriaxone/Linezolid	Chronic spinal osteonyelitis	No clinical utility
73	Spine tissue - Spinal rod	Vancomycin/Cefepime	No growth but Group B *Streptococcus* in blood culture	No bacterial DNA detected	Ampicillin	Paraspinal abscess	No clinical utility
74	Spine tissue - Spinal rod biopsy	Vancomycin/Cefepime	No growth	No bacterial DNA detected	Cefepime/Linezolid	Cerebrospinal Fluid leak/presumed meningitis	No clinical utility
